# Elements of Dental Materia Medica and Therapeutics

**Published:** 1878-01

**Authors:** 


*Elements of Dental Materia Medica and Therapeutics. with Pharmacopœia.*—By James Stocken, L. D. S., R. C. S., Eng. Lecturer on Dental Materia Medica and Therapeutics at the National Dental College, London, Etc. Etc. Publishers: J. and A. Churchhill, London, England, 1877.

This is an elementary treatise similar in style, form and size, to a work edited by Jas. W. White, D. D. S., and published some years ago by S. S. White, of Philadelphia, under the name of Dental Materia Medica, with such additions, however, in the English work as a dental pharmacopœia, antidotes to the principal poisons, definitions of terms denoting the properties of Remedial Agents, and tables of approximate measurement, pulsation per minute at various ages, also respiration and doses for adult males, females and children. While there is much useful information in this work for the dental practitioner and student, yet it is too elementary in its character to be adopted as a complete text book in dental colleges, where more of the phyiological effects, chemical constituents, and medicinal uses, as well as dental uses, of the different agents composing dental materia medica should be taught, than is contained in this small work. Its size does not admit of the notice of quite a number of the agents now employed therapeutically in dental practice. Lindsay and Blakiston, Philadelphia, are the American publishers.

---

